# Genetic Influences on Incidence and Case-Fatality of Infectious Disease

**DOI:** 10.1371/journal.pone.0010603

**Published:** 2010-05-14

**Authors:** Liselotte Petersen, Per Kragh Andersen, Thorkild I. A. Sørensen

**Affiliations:** 1 National Centre for Register-Based Research, University of Aarhus, Aarhus, Denmark; 2 Institute of Preventive Medicine, Copenhagen University Hospital, Copenhagen, Denmark; 3 Department of Biostatistics, University of Copenhagen, Copenhagen, Denmark; Innsbruck Medical University, Austria

## Abstract

**Background:**

Family, twin and adoption studies suggest that genetic susceptibility contributes to familial aggregation of infectious diseases or to death from infections. We estimated genetic and shared environmental influences separately on the risk of acquiring an infection (incidence) and on dying from it (case fatality).

**Methods:**

Genetic influences were estimated by the association between rates of *hospitalization for infections* and between *case-fatality* rates of adoptees and their biological full- and half- siblings. Familial environmental influences were investigated in adoptees and their adoptive siblings. Among 14,425 non-familial adoptions, granted in Denmark during the period 1924–47, we selected 1,603 adoptees, who had been *hospitalized for infections* and/or *died with infection* between 1977 and 1993. Their siblings were considered *predisposed to infection*, and compared with *non-predisposed* siblings of randomly selected 1,348 adoptees alive in 1993 and *not hospitalized for infections* in the observation period. The risk ratios presented were based on a Cox regression model.

**Results:**

Among 9971 identified siblings, 2829 had been *hospitalised for infections*. The risk of *infectious disease* was increased among *predisposed* compared with *non-predisposed* in both biological (1.18; 95% confidence limits 1.03–1.36) and adoptive siblings (1.23; 0.98–1.53). The risk of a *fatal outcome* of the infections was strongly increased (9.36; 2.94–29.8) in biological full siblings, but such associations were not observed for the biological half siblings or for the adoptive siblings.

**Conclusion:**

Risk of getting infections appears to be weakly influenced by both genetically determined susceptibility to infection and by family environment, whereas there appears to be a strong non-additive genetic influence on risk of fatal outcome.

## Introduction

Using current genomic technology, there is an intense ongoing search for specific genes in which variations influence host susceptibility to infectious diseases [1]–[4]. Although some evidence suggests that there is a genetic specificity in host-infectious disease association (one gene and multiple different infections; multiple genes and one infection; and, recently, one gene and one infection) [5], it seems plausible that there may also be a polygenic influence on the general susceptibility to infection irrespective of type of infection [6]–[9]. Studies of aggregation of infections in families will obviously have great difficulties in disentangling effects of shared exposure to the infectious agent from common underlying genetic susceptibility. However, some studies of the familial occurrence of acute meningococcal infections have found that the time lag between the events is so long that it is very unlikely that shared exposure to the infectious agents plays a role in the familial aggregation. Assuming that lasting environmental influences can be excluded, this suggests that a genetically determined susceptibility may be the reason for the aggregation [10]. Analysis of genealogies combined with death certificates in the Utah population over 100 years, came to the same conclusion for death from influenza [11]. However, not all studies agree; a recent very large-scale study from Denmark, utilizing access to nation-wide registers on invasive pneumococcal infections, only found an increased familial risk during the time period of likely shared exposures [12]. Heterogeneity in the phenotype in terms of severity and eventual outcome of the infections, including fatal outcome, may explain the different results.

Twin studies may help distinguishing the role of shared environment, including shared exposure, and genetic effects [9], [13]–[18], but only on the assumption of equal shared environmental effects in mono- and dizygotic pairs, especially for the contagious exposures, which, however, may be a dubious assumption, because monozygotic twins see each other more frequently than dizygotic twins [19]. The problem of possible confounding of the genetic influence by the shared environment may be solved by studying the aggregation of infections in adoptees and in their biological relatives, who the adoptee have been effectively separated from since birth or shortly thereafter. Thus, neither the biological full nor the biological half siblings did live in the same household as their adopted away sibling. We have conducted studies of cause-specific mortality in various subsets of an adoption cohort defined by adoptions taking place from 1924 through 1947, and have found evidence for a genetic influence on risk of dying with infection [20]–[23].

We suggest that the results indicate that genes influencing the risk of acquiring an infection may be different from genes affecting the course of the disease. Using the abovementioned adoption cohort, we have therefore made an assessment of the genetic and shared environmental influences separately on the risk of acquiring an infection (incidence) and on dying from it (case fatality).

## Methods

### Sampling for the analyses of incidence infections

**Figure 1:**
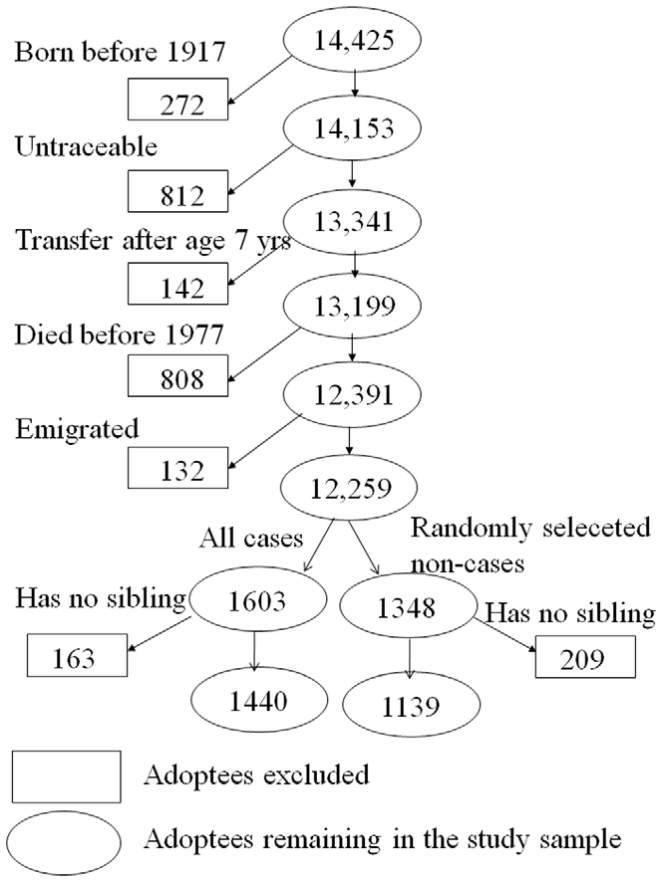
Flow chart illustrating the number of adoptees in the study population.

The study was based on the Danish Adoption Register, which contains records on all non-familial adoptions formally granted in Denmark during the period 1924 through 1947 [20], [24], [25]. We included adoptees born in 1917 or later who were traceable, were transferred to the adoptive family before the age of 7 years, and were alive in 1977 (See flowchart in Figure 1 for details). There were 12,391 such adoptees of whom 1,603 had been *hospitalized for infection* between 1 Jan 1977 and 1 April 1993 or who *died with infection*, which hence was assumed to be a contributing cause of death. Siblings to these adoptees are defined as *predisposed to infection*. From the 10,788 non-case adoptees we selected a random sample of 1,480. In this random sample 132 who died or emigrated before 1 April 1993 were excluded, as they were not at risk of becoming cases as long time as the others. Siblings to the remaining 1348 adoptees are defined as *non-predisposed to infection*. The study sample therefore consists of the 2579 ( = 1440+1139) adoptees for whom it was possible to trace adoptive and/or biological siblings in the regionally organized civil registers.

**Figure 2:**
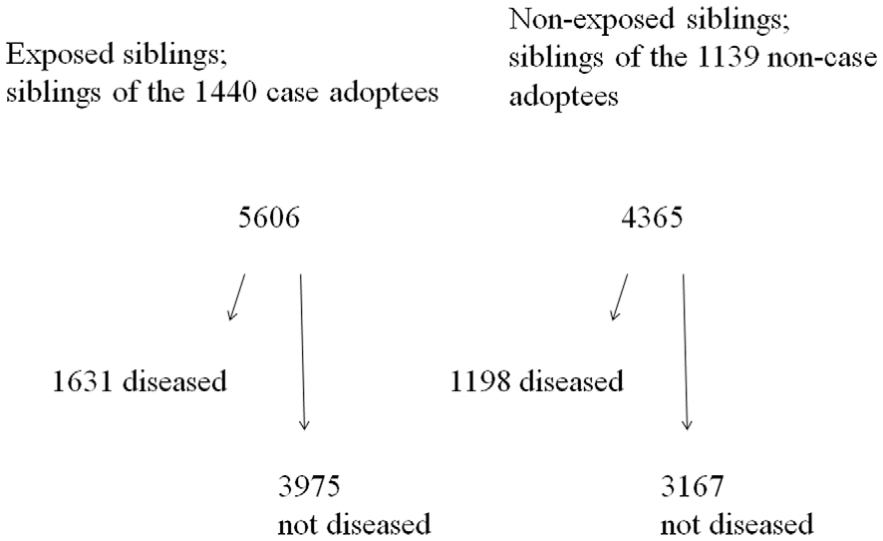
The 9971 siblings in the analyses of incidence.

The siblings were identified by the record linkage in our population registers for each person of his or her parents and his or her children. All together, 9,994 siblings were alive in 1977 and not above 70 years (1465 died earlier than 1977, and 33 persons were above 70 years in 1977). Of the selected adoptees 23 were adopted with their twin; these twin siblings were excluded because they could count neither as full siblings nor as adoptive siblings, and they were too few to justify a separate twin model. The remaining 9971 siblings were followed either until a *hospitalization with infection* as discharge diagnoses, until *death with infection*, or until censoring at 31 December 2006, whichever came first. Figure 2 presents the siblings according to their disease status and the disease status of the adoptees. In Table 1 the siblings are further divided into biological full- and half siblings and adoptive siblings. Medians and ranges of ages of biological and adoptive siblings at start of follow-up are indicated in Table 1.

**Table 1:** The 9971 siblings according to their own disease status and by disease status of the adoptee (1440 diseased adoptees and 1139 not diseased adoptees).

Siblings		Diseased^1^	Not diseased	Median age (range)^2^	
Full siblings	Diseased adoptee	224	465		
	Not diseased adoptee	148	370	37	(10–70)
Paternal half siblings	Diseased adoptee	605	1507		
	Not diseased adoptee	429	1174	32	(0–70)
s Maternal half siblings	Diseased adoptee	590	1434		
	Not diseased adoptee	489	1221	32	(7–70)
Adoptive siblings	Diseased adoptee	212	569		
	Not diseased adoptee	132	402	35	(10–70)
In total		2829	7142		

1 The diseased are hospitalized with infection or with infection as a cause of death.

2 Age at baseline in 1977.

### Sampling for the analyses of case-fatality

**Figure 3:**
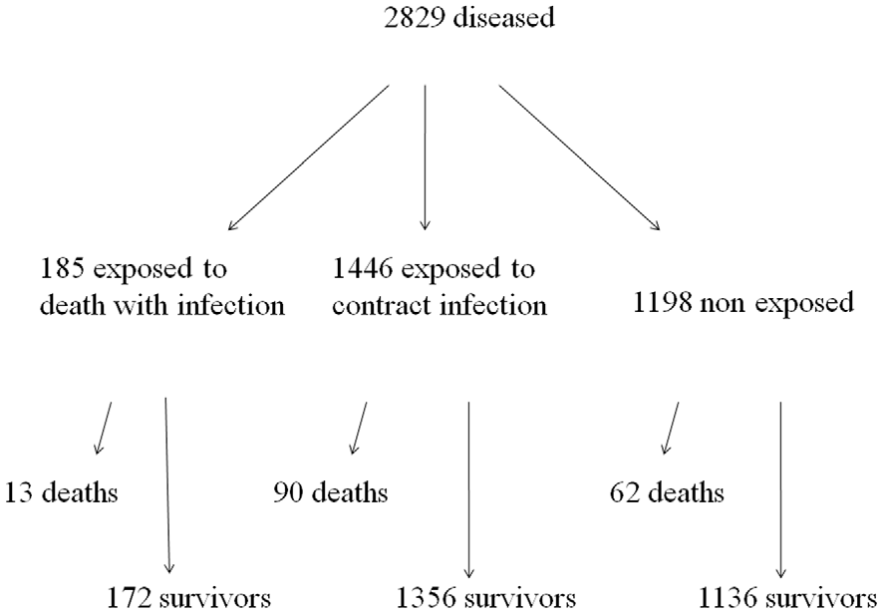
The 2829 (1631+1198) diseased siblings in the analyses of case-fatality.

To examine case fatality, all infected siblings were analysed using *death with infection* recorded as possible cause of death as outcome. The selection of this sample, within the 9971 siblings, is based on the disease status of the siblings, independent of the status of the adoptee. I.e. from the case non-case sample, the adoptees included are those having at least one sibling *admitted with an infectious disease*, which counts 1620 adoptees. For these analyses the *nonpredisposed* are the 1198 siblings to non-case adoptees, whereas the *predisposed* were divided in two; one cohort *predisposed to death with infection* consisting of 185 siblings to an adoptee who *died with infection* between 1 January 1977 and 1 April 1993, and another cohort of *predisposed to contract infection* consisting of 1446 siblings to an adoptee who were *hospitalized with infection* between 1 January 1977 and 1 April 1993. Figure 3 presents the number of siblings *dying with infection* among those infected according to the disease status of the adoptee. In Table 2 the siblings are further divided into biological full- and half siblings and adoptive siblings. Medians and ranges of ages of biological and adoptive siblings at time of admission with infection are indicated in Table 2.

**Table 2:** The 2829 diseased siblings divided into deaths and survivors by disease status of the adoptee (95 adoptees deaths with infections, 827 diseased adoptees and 698 not diseased adoptees).

Siblings		Deaths	Survivors	Median age (range)^1^	
Full siblings	Adoptee died with infection	5	16		
	Adoptee diseased	16	187		
	Adoptee not diseased	9	139	53	(17–70)
Paternal half siblings	Adoptee died with infection	4	68		
	Adoptee diseased	28	505		
	Adoptee not diseased	25	404	49	(17–70)
Maternal half siblings	Adoptee died with infection	3	67		
	Adoptee diseased	30	490		
	Adoptee not diseased	18	471	49	(16–70)
Adoptive siblings	Adoptee died with infection	1	21		
	Adoptee diseased	16	174		
	Adoptee not diseased	10	122	51	(18–70)
In total		165	2664		

1 Age at admission with infection.

### Follow-up and ascertainment of disease status

Biological and adoptive siblings were followed until death or 31 December 2006. From the national hospitalization register, we extracted information about hospital admission of which the discharge diagnoses included any diagnoses known or assumed very likely to be caused by an infection according to the international disease classification systems in use at the time of the diagnosis. The cause of death was recorded irrespective of its position on the death certificate.

### Statistical methods

The study design can be seen as two separate cohorts, one cohort *predisposed to infection* and one cohort *non-predisposed to infection*. The rates of *hospitalisation due to infection* before age 70 years of siblings *predisposed* or *non-predisposed* were compared in a Cox regression model with age as time variable by including status of the adoptee indicating predisposition group as explanatory variable.

Among 2829 diseased siblings, 55 *died with infection* without prior *hospitalization with infection* and 3 died on the day of admission; these 58 were given a risk time of half a day. Among the siblings, 14 died during hospitalization, i.e. they had a risk time equal to the number of days at the hospital. The rates of *death with infection* before age 70 years of the various groups of siblings were compared in a Cox regression model with age as time variable by including status of the adoptee indicating predisposition group as explanatory variable.

The Cox models were stratified by sex of the siblings, and by categories of year of birth (five categories for adoptees, and up to three categories for siblings). The proportional hazards assumptions were tested by Schoenfelds residuals and were not rejected. All siblings, biological full and half siblings and adoptive siblings were included in the same model. Siblings of the same adoptee comprised a cluster, and allowance for a possible within-cluster dependence was made by using robust standard error estimates. Full siblings, on average, have twice as many genes in common as half siblings, so a possible genetic effect in full siblings may be equal to twice the effect in half siblings. Although this will be scale-dependent and requires additive genetic effects on this scale, we decided to test this relation on the log hazard ratio scale. On the hazard ratio (HR) scale this means that the HR in full siblings equals the HR in half siblings squared. Analyses were carried out using Stata version 9.0 (StataCorp, College Station, TX). We calculated HRs and 95% confidence intervals (CIs).

### Ethics

Since the study is entirely based on register data, there was according to Danish law no request for an ethical permission. The study was approved by Danish Data Protection Agency.

## Results

Table 3 presents associations of *predisposition to infection* with rate of *hospitalisation due to infection* among siblings. The associations for paternal and maternal half siblings were not statistically significantly different (p = 0.55). The hypothesis that the effects in half siblings are half the effects in full siblings (on the log HR scale) was not rejected (p = 0.50). As seen in Table 3, we found an excess HR (1.26; 95% confidence interval (CI) of 1.01–1.57) for *hospitalisation due to infections* of *predisposed* full siblings compared with *non-predisposed* full siblings. For all biological siblings combined, the HR was (1.18; 1.03–1.36). In the analyses of adoptive siblings, rates of *hospitalisation for infection* were increased among those being an adoptive sibling to an adoptee *hospitalised with infection*, and the HR was borderline statistically significant, (1.23; 0.98–1.53).

**Table 3:** Rate of hospitalization due to infectious disease and rate of death with infection among biological and adoptive siblings, in *predisposed* siblings compared to *non-predisposed*.

HR (95% CI)	Hospitalization of siblings	Mortality of diseased siblings	
	Given predisposition to infection^1^	Given predisposition to contract infection^2^	Given predisposition to death with infection^3^
Full siblings	1.26 (1.01–1.57)	1.76 (0.78–3.98)	9.36 (2.94–29.8)
Paternal half siblings	1.10 (0.97–1.25)	1.18 (0.65–2.14)	0.78 (0.21–2.96)
Maternal half siblings	1.04 (0.92–1.18)	1.19 (0.63–2.28)	0.94 (0.27–3.33)
Biological siblings combined^4^	1.18 (1.03–1.36)	1.53 (0.86–2.74)	-5
Adoptive siblings	1.23 (0.98–1.53)	1.12 (0.50–2.53)	0.76 (0.06–9.87)

Analyses are stratified by sex of adoptee and sibling, and by birth year group of adoptee and sibling. Siblings of the same adoptee are analysed in one model allowing for correlation between observations.

1 Siblings to an adoptee hospitalized with infection or with infection as a cause of death.

2 Siblings to an adoptee hospitalized with infection.

3 Siblings to an adoptee with infection as a cause of death.

4 Assuming additive genetic effect on the log hazard scale.

5 Assuming additive genetic effect is inappropriate.

Death with infection as outcome occurred in 165 siblings before age 70 years. Table 3 presents the rate of mortality with infections as presumed cause among diseased siblings. *Predisposition to infection* are divided into *predisposition to contract infection* and *predisposition to death with infection*, according to the disease status of the adoptee. Being a full sibling to an adoptee who died with infection increased the mortality rate with infection to a highly significant HR of (9.36; 2.94–29.8, p = 0.0001), whereas HRs among half siblings were not statistically different from one. The relative size of the estimates gave no support to an additive genetic model, and the hypothesis that effects in half siblings are half the effect in full siblings on the log HR scale was rejected (p = 0.02).

To investigate the consistency of the main findings, we conducted a series of supplementary analyses described in the following (results are not shown). Restricting the follow-up period among siblings to April 1993 (the end of follow-up of the adoptees), and onwards reduced the number of events considerably, i.e. to 1385 out of 2829 hospitalizations for infections and 84 out of 165 deaths with infection, but the pattern of results was unchanged. Some siblings had more than one sibling being adopted away, and are therefore represented more than once in our dataset; keeping only one (randomly chosen) observation of each person, left out 159 observations, but this did not change the estimates. No modifying effect of gender of the siblings was found (p
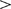
0.24). The siblings were divided into three groups according to birth cohort, but no modification of the associations was found.

## Discussion

This study showed that the rate of getting a severe infectious disease, requiring hospitalization, is increased by about 20% in both biological and adoptive siblings of adult adoptees who themselves have suffered from such disease. These findings suggest that the risk of acquiring infections requiring hospitalizations is influenced by both a genetic susceptibility and the environment shared in the family. This interpretation rests on the assumption that the observed association is generated only by the genes in common between adoptees and their biological siblings and only by the shared environment among the adoptees and the adoptive siblings, respectively. The study also showed significantly increased risk for biological full siblings and a less, but not significantly increased and not significantly different, risk for biological paternal or maternal half siblings; this pattern is compatible with genetic interpretation of additive nature.

The risk of a fatal outcome of the infectious disease was greatly increased in biological full siblings to adoptees who died from their infection, but such associations were not observed for the biological half siblings or for the adoptive siblings. This finding suggests that there is a strong genetic influence on the risk of dying from the infections, and that this influence is non-additive. This pattern may arise from a mutual dependency of gene variants (recessive or epistatic effects), which are likely to be transmitted together only among full siblings. Our findings are compatible with previously reported twin studies [8], [9], [11], [13]–[17], although these studies did not distinguish between incidence and case-fatality and, in any case, would leave doubt about whether the influence of shared environment, especially exposure to the microbes, have been similar in the two types of twin pairs.

The interpretation of the outcome of adoption studies of siblings, like the present one, rests on a series of assumptions [20]–[23], [25]. The most fundamental assumption of the genetic interpretation is that the shared environmental influences during the early pre- and postnatal life of the adoptees and their biological siblings until separation from the biological family is irrelevant for the associations of risk of diseases among them many years later. If such early environmental influences are operating, they may generate associations that are similar to those possibly generated by the genetic relatedness, including the stronger association between full siblings than between half siblings, whether additive or non-additive. Paternal half siblings may have the least influence of early shared environment, and observing the association at interest in this group may corroborate the genetic interpretation, but this is particularly demanding because of the dilution of the genetic relatedness, partly because of the half sibling status (on average only 25% of the genome is the same in the sibling pairs) and partly because of possible non-paternity. In the present study, we did not find significant differences between maternal and paternal half siblings, but statistical power to make such distinction is rather limited.

The ability of the adoption study to separate genetic and environmental influences on any phenotype requires that the adoptees have been placed in an adoptive family that is genetically unrelated to the biological family. The adoption cohort used in the present study was carefully screened for any relationship between the biological and the adoptive family. It seems very unlikely that the selection process of the adoptive family in any way is influenced by the phenotype under study here with the risk of infections emerging many years after the adoption. For the genetic interpretation, it is similarly assumed that there has been no or only irrelevant contact between the adoptees and the biological siblings, and previous thorough investigation of the contacts between the families in a subset of the cohort support that this should not be a problem [26].

The interpretation of finding of the association in risk of infections between adoptee and their adoptive siblings as based on shared environmental exposures appears straightforward, although the study does not allow a distinction between shared environmental influences on susceptibility to infection, e.g. by shared living conditions during childhood, and on exposure to the infectious agent. Investigation of the effect of the time interval on the strength of the association, used in some previous studies of familial aggregation of infections [10], [12], may help show whether the particular disease events are related, but may not help in disentangling the two modes of environmental influences, possibly operating whether the time intervals are short or long.

In our previous studies of all-cause and cause-specific mortality in the same adoption cohort [20]–[23], we have found that there is evidence suggesting genetic influences on mortality from infections, and no or little influence of the environment shared with the adoptive parents. In the studies of parent-offspring pairs, genetic effects on mortality with infections were found in the earliest birth cohorts of adoptees [20]–[22]. In a study of adoptees and their siblings, belonging to a sample that is part of this study, evidence for a genetic effect on mortality from infection was seen in all birth cohorts [23].

These findings lead to questions about whether the supposed genetic influence is operating on the risk of diseases incidence and/or of case fatality. Since we have no access to systematic information about morbidity until 1977 (the year of launching the national hospital register), the question cannot be addressed as a parent-offspring study for a relevant age period in this cohort. This limitation further implies that the results pertain to the adoptees from 30 through 70 years of age and their siblings from 0 through 70 years. Each person is followed for a maximum of 40 years; defined by the time the register has existed. Consequently, some siblings are followed just a few years and thereby probably caused some false negative observations, leading to a non-differential misclassification. No adoptees and only some of the siblings were followed during childhood. If genetic influences are strongest early in life, this limitation is likely to have resulted in conservative estimates.

In view of the findings in other settings of gender differences in infectious disease mortality and reactions to vaccinations [27], it is noteworthy that neither our former studies nor the present study showed any gender differences.

Grouping all infections together, regardless of type of infection, implicitly restricts a possible interpretation of a genetic finding to be general across types of infections possibly polygenetic influence. However, the strong genetic association for mortality with infection suggests that even such a crude measure may be appropriate. Further, as the information on causes of death is gathered from the death certificates, some misclassification cannot be ruled out; however, this is likely to cause non-differential misclassification.

The interpretation of our findings as well as those reported in the literature on possible genetic susceptibility raise the question of which aspect of the infectious disease phenotypes the genes are affecting. The finding of the apparently strong genetic influence on case-fatality in our study suggests that the genetic susceptibility is operating in the defence against progression of the infection rather than acquirement of the infection. Our measure of the latter phenotype is based on hospitalization for the infectious disease; in itself this may also involve a component of clinical severity of the disease, which may be behind the observed slightly elevated risks of getting the infectious diseases. This feature of the phenotype, ‘the severity of infectious disease’, may contribute to explain the discrepancies between the results obtained in the recently reported study of a large-scale, nation-wide, population-based Danish cohort [12] and our and other previous studies. The Danish cohort study showed that the occurrence of invasive pneumococcal infection was not associated with increased risk of the same sort of invasive infections among 1st, 2nd and 3rd degree relatives, not sharing the household around the time of the index case of invasive pneumococcal disease, but the study did not address severity of disease or case-fatality [12]. The results of studies of the phenotype, severe infectious disease, are obviously dependent on thresholds for hospital admission and whether the infections investigated at the time and place they occurred were successfully treated. The continuous improvement in hospital care and treatments available for severe infections may be a reason why the older studies show greater associations suggesting genetic influences than the more recent ones.

The biological mechanisms by which individual genetic variation may influence the susceptibility to infectious disease and especially the outcome of the disease are likely to be complex and involve multiple different pathways as also suggested by the ongoing research using the genome-wide association studies of single nucleotide polymorphisms [1]–[9], [28]. So far, this new approach has revealed several new genes of possible importance, but there is also, as in other fields of complex disease mechanisms, an emerging recognition that this technology may not be able to identify the entire molecular genetic basis for the quantitatively demonstrated genetic susceptibility. Our results suggest that in the search for the underlying genes, it may be helpful to specify the aspects of the rather broadly defined phenotype, infection, and focus particularly on measures of the severity and outcome of the infection. The finding of a non-additive pattern of the genetic influence may also help in the search for the molecular genetic basis by specifying that the expectations of genotype-phenotype associations may be much greater if allele-allele and gene-gene interactions are properly taken into account in designing the studies and analysing the data. Successful identification of the molecular genetic basis for the non-additive genetic influence on outcome of the infections may aid the development of improved modalities for prevention and treatment of these diseases.

## References

[1] Tuite A, Gros P (2006). The impact of genomics on the analysis of host resistance to infectious disease.. Microbes and Infection.

[2] Motsinger AA, Haas DW, Hulgan T, Ritchie MD (2007). Human genomic association studies: a primer for the infectious disease specialst.. JID.

[3] Xavier RJ, Rioux JD (2008). Genome-wide association studies: a new window into immune-mediated diseases.. Nature Rev Immunol.

[4] Brouwer MC, de Gans J, Heckenberg SGB, Zwinderman AH, van der Poll T (2009). Host genetic susceptibility to pneumococcal and meningococcal disease: a systematic review and meta-analysis.. Lancet.

[5] Casanova JL, Abel L (2007). Human genetics of infectious diseases: a unified theory.. EMBO Journal.

[6] Kwaitkowski D (2000). Susceptibility to infections.. BMJ.

[7] Hill AVS (2001). The genomics and genetics of human infectious disease susceptibility.. Annu Rev Genomics Hum Genet.

[8] Cooke GS, Hill AVS (2001). Genetics of susceptibility to human infectious disease.. Nature Rev Genetics.

[9] Burgner D, Jamieson SE, Blackwell JM (2006). Genetic susceptibility to infectious diseases: big is beautiful, but will bigger be even better?. Lancet Infect Dis.

[10] Haralambous E, Weiss HA, Radalowicz A, Hibberd ML, Booy R (2003). Sibling familial risk ratio of meningococcal disease in UK Caucasians.. Epidemiol Infect.

[11] Albright FS, Orlando P, Pavia AT, Jackson GG, Albright LAC (2008). Evidence for a heritable predisposition to death due to influenza.. JID.

[12] Hjuler T, Poulsen G, Wohlfahrt J, Kaltoft M, Biggar R (2008). Genetic susceptibility to severe infection in families with invasive pneumococcal disease.. Am J Epidemiol.

[13] Comstock GW (1993). Tuberculosis in twins: a re-analysis of the Prophit survey.. Am Rev Respir Dis.

[14] Lin TM, Chen CJ, Wu MM, Yang CS, Chen JS (1989). Hepatitis B virus markers in Chinese twins.. Anticancer Res.

[15] Malaty HM, Engstrand L, Pedersen NL, Graham DY (1994). Helicobacter pylori infection: genetic and environmental influences – a study of twins.. Ann Int Med.

[16] Casselbrant ML, Mandel EM, Fall PA, Rockette HE, Kurs-Lasky M (1999). The heritability of otitis media: a twin and triplet study.. JAMA.

[17] Rovers M, Haggard M, Gannon M, Koeppen-Schomerus G, Plomin R (2002). Heritability of symptom domains in otitis media: a longitudinal study of 1,373 twin pairs.. Am J Epidemiol.

[18] Obel N, Christensen K, Petersen I, Sørensen TIA, Skytthe A (2010). Genetic and environmental influences on risk of deaths due to infections assessed in danish twins, 1943–2001.. Am J Epid Epub.

[19] Lykken DT, McGue M, Bouchard TJJ, Tellegen A (1990). Does contact lead to similarity or similarity to contact?. Behav Genet.

[20] Sørensen TIA, Nielsen GG, Andersen PK, Teasdale TW (1988). Genetic and environmental influence on premature death in adult adoptees.. N Engl J Med.

[21] Petersen L, Nielsen GG, Andersen PK, Sørensen TIA (2002). Case-control study of genetic and environmental influences on premature death of adult adoptees.. Genetic Epidemiol.

[22] Petersen L, Andersen PK, Sørensen TIA (2005). Premature death of adult adoptees, analyses of a case-cohort sample.. Genetic Epidemiol.

[23] Petersen L, Andersen PK, Sørensen TIA (2008). Genetic and environmental effects on the rate of dying before age 70 years.. Epidemiol.

[24] Kety SS, Rosenthal D, Wender PH, Schulsinger F (1968). The types and prevalence of mental illness in the biological and adoptive families of adopted schizophrenics.. J Psychiatr Res.

[25] Borch-Johnsen K, Sørensen TIA (1993). Genes and environment in the inheritance of morbidity and mortality.. Acta Psychiatr Scand Suppl.

[26] Eldred CA, Rosenthal D, Wender PH (1976). Some aspects of adoption in selected samples of adult adoptees.. Am J Orthopsychiatry.

[27] Aaby P (2007). Is susceptibility to severe infection in low-income countries inherited or acquired.. J Intern Med.

[28] Westendorp RG, Langermans JA, Huizinga TW, Eloubali AH, Verweij CL (1997). Genetic influence on cytokine production and fatal meningococcal disease.. Lancet.

